# Differential expression and co-expression gene network analyses reveal molecular mechanisms and candidate biomarkers involved in breast muscle myopathies in chicken

**DOI:** 10.1038/s41598-019-51521-1

**Published:** 2019-10-17

**Authors:** Eva Pampouille, Christelle Hennequet-Antier, Christophe Praud, Amélie Juanchich, Aurélien Brionne, Estelle Godet, Thierry Bordeau, Fréderic Fagnoul, Elisabeth Le Bihan-Duval, Cécile Berri

**Affiliations:** 1 BOA, INRA, Université de Tours, 37380 Nouzilly, France; 2 Hubbard SAS, Mauguérand, 22800 Le Foeil - Quintin, France

**Keywords:** Gene expression analysis, Transcriptomics

## Abstract

The broiler industry is facing an increasing prevalence of breast myopathies, such as white striping (WS) and wooden breast (WB), and the precise aetiology of these occurrences remains poorly understood. To progress our understanding of the structural changes and molecular pathways involved in these myopathies, a transcriptomic analysis was performed using an 8 × 60 K Agilent chicken microarray and histological study. The study used *pectoralis major* muscles from three groups: slow-growing animals (n = 8), fast-growing animals visually free from defects (n = 8), or severely affected by both WS and WB (n = 8). In addition, a weighted correlation network analysis was performed to investigate the relationship between modules of co-expressed genes and histological traits. Functional analysis suggested that selection for fast growing and breast meat yield has progressively led to conditions favouring metabolic shifts towards alternative catabolic pathways to produce energy, leading to an adaptive response to oxidative stress and the first signs of inflammatory, regeneration and fibrosis processes. All these processes are intensified in muscles affected by severe myopathies, in which new mechanisms related to cellular defences and remodelling seem also activated. Furthermore, our study opens new perspectives for myopathy diagnosis by highlighting fine histological phenotypes and genes whose expression was strongly correlated with defects.

## Introduction

The poultry industry relies on the production of fast-growing chickens, which are slaughtered at high weights and intended for cutting and processing. It is currently facing severe myopathic defects, such as white striping (WS), which is characterized by the appearance of lipid and connective inclusions that develop parallel to the muscle fibres^1–3^, or wooden breast (WB), which is characterized by a heterogeneity of colour, excessive surface exudate and a loss of muscle elasticity^4,5^. These two defects significantly impair breast fillet appearance and therefore consumer purchases^6^. Compared to normal fillets, these defects also affect meat nutritional value by increasing fat and connective tissue content and lowering muscle protein content^7–11^ and water holding capacity (WHC), resulting in higher drip (DL) and cooking (CL) loss, lower curing cooking yield (CCY) and marinade uptake^3,5,11,12^. Breast fillets that are severely affected by WS or WB are difficult to market fresh and are more often valued as raw materials for processing or downgraded for pet food in the most severe cases^13^. Therefore, these defects lead to significant economic losses for the poultry industry. Moreover, an increasing incidence of muscular defects is likely to degrade the image of chicken meat by consumers, as attested by the increasing number of articles dedicated to the subject in the specialized or general press.

Although myodegenerative defects are now widely described in the literature^1–4,14^, their precise aetiology remains unclear. What is certain is that the frequency and severity of these defects increase with the growth rate of the animals, their slaughter weight and their breast meat yield (BMY)^3,7,15^. In addition, their increasing occurrence corresponds to changes in current production practices, particularly the use of increasingly efficient genotypes and the slaughter of heavy broilers, whose meat is intended for processing. The genetic basis of WS and WB has been recently demonstrated^16,17^, but the genes involved in the control of these defects are still unknown despite the recent identification of QTL, which controls WS^18^. In one study several chromosomal regions of interest in WS were suggested, and the results indicated a polygenic determinism for the trait and include a list of putative candidate genes that primarily affect muscle metabolism, fibre structure and human neuromuscular disorders^18^. Therefore, improving understanding of the molecular mechanisms that underlie muscle susceptibility to WS and WB remains an important objective from the perspective of more efficient selection and improved breeding conditions.

Recent transcriptomic studies have described changes in muscle gene expression related to the occurrence and severity of myodegenerative defects measured in fast-growing broilers^19–23^.

In the current study, we compared the gene expression profile of the *pectoralis major* muscle between birds obtained from a slow-growing chicken line (SG) and birds obtained from a modern fast-growing line; all birds were visually scored as either severely affected by both WS and WB defects (FG-WSWB) or free from defects (FG-C). We further included a slow-growing line in which no defect was observed with the aim of identifying the biological changes induced by long-term selection on body weight and muscle development that has resulted in the establishment of the myopathies. This study is also focused on the search for fine and histological traits that can be used to quantify muscular defects with the aim of correlating them with gene expression and identifying biomarkers of myopathic muscles.

## Results

### Animals and descriptive statistics of meat quality phenotypes

The transcriptomic analysis was performed on 3 × 8 individuals obtained from the SG, FG-C and FG-WSWB groups. FG individuals were obtained from a grand-parental population of 176 42-day-old broilers visually scored for WS and WB. Individuals were chosen to be representative of either the control (i.e., without any apparent defects) or the severely affected (i.e., affected with both the WS and WB defects) categories. SG birds were obtained from a slow-growing INRA experimental line that was also slaughtered at 42 days of age. Only body weight (BW) and *pectoralis major* yield (PMY) were measured in this line. Student’s t-test revealed that BW at 42 days of age was more than three times higher (3324 vs 999 g; p-value ≤ 0.0001) and PMY was 25% higher (17.9 vs 13.5%; p-value ≤ 0.0001) in fast-growing broilers than in slow-growing birds.

The average phenotype values of the FG-C and FG-WSWB groups are described in Supplementary Table S1. FG-C and FG-WSWB chickens showed similar BW and abdominal fatness (AFP). BMY did not differ between FG-WSWB and FG-C chickens (p-value = 0.07), while the difference between the groups was significant when considering only PMY (p-value ≤ 0.05). FG-WSWB breast muscles exhibited much greater DL and CL (p-value ≤ 0.001 and p-value ≤ 0.01, respectively) and tended to have lower CCY and higher lightness (L*) (p-value = 0.07) than were observed in FG-C chickens. They did not differ in other meat quality traits, including lipid peroxidation index (evaluated through TBA-RS) after storage and shear force (SF) value after cooking.

### Fine and quantitative histological traits

**Figure 1:**
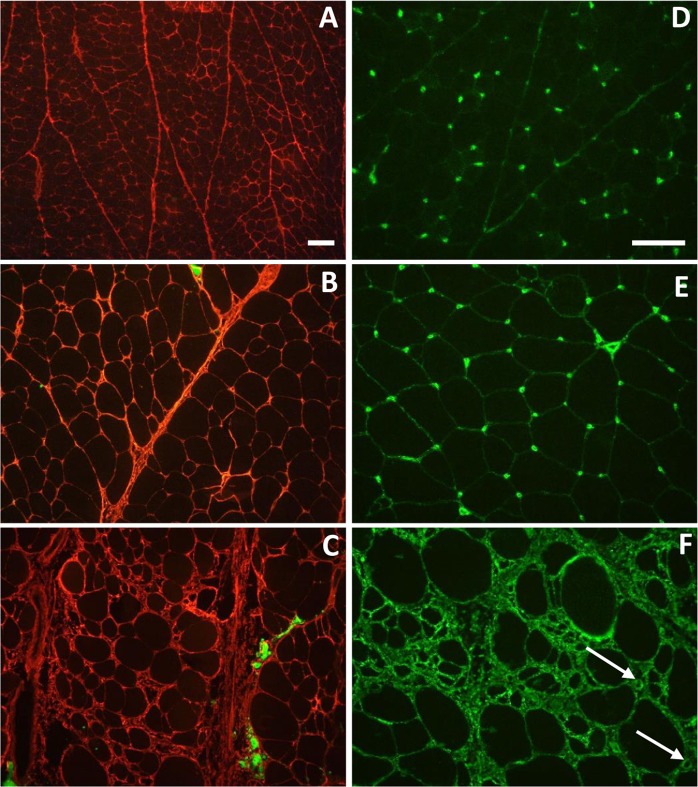
Collagen VI-Bodipy co-labelling (**A–C**) and fibronectin labelling (**D**–**F**) fluorescence micrographs of SG (**A,D**), FG-C (**B,E**) and FG-WSWB (**C,F**) *pectoralis major* muscles. Collagen VI labelling is in red and bodipy in green (**A–C**). Capillaries in the affected muscles (**F**) are indicated by arrows. Scale bar: 100 µm. A,B, and C: 10× objective, D,E and F: 20× objective.

To quantify muscular defects, quantitative histological traits were measured in *pectoralis major* muscle cross sections obtained from the three groups (SG, FG-C and FG-WSWB) (Table 1). A quantification of fibrosis and adiposis was performed using a collagen VI-bodipy co-labelling technique (Fig. 1A–C). The percentage of the area labelled with collagen VI (representative of fibrosis) on the microscopic field was 4.2 and 6.9 times higher in FG-C and FG-WSWB muscles, respectively, than in SG *pectoralis major* muscles (p-values ≤ 0.0001). Thus, compared to SG muscles, both FG-C and FG-WSWB muscles showed extended endomysial and perimysial connective tissues, but the rate was 1.6 lower in the FG muscles macroscopically unaffected by WS and WB than in affected muscles. It is interesting to note that the percentage of the area labelled with collagen VI in one sample of the FG-WSWB group and one sample of the FG-C group was very different than the average value obtained in their respective groups, i.e., it was lower for the FG-WSWB sample (8.1%) and higher for the FG-C sample (13.6%). This suggests that these samples were either misclassified macroscopically or phenotypically intermediate between the FG-C and the FG-WSWB classes. However, the percentage of the area occupied by bodipy 493 staining (representative of adiposis) was approximately three times larger in FG-WSWB than in FG-C *pectoralis major* muscles (p-value ≤ 0.01) and was almost non-existent in SG *pectoralis major* muscles. These results indicate that muscle histological architecture is modified according to the selection on growth rate and that fibrosis and adiposis establishment are exacerbated in cases of severe defects.

**Table 1:** Means and standard deviations for histological traits measured in *pectoralis major* muscle cross-sections.

Traits	SG (N = 8)	FG-C (N = 8)	FG-WSWB (N = 8)	p-value
Collagen VI,%^*^	2.13 ± 0.73^a^	9.06 ± 2.96^b^	14.73 ± 4.14^c^	1.58 × 10^−7^
Adipose tissue, %^*^	0.05 ± 0.12^a^	0.36 ± 0.27^a^	1.10 ± 0.82^b^	1.29 × 10^−3^
Number of fibres per field	203.7 ± 21.2^a^	51.1 ± 11.4^b^	45.1 ± 9.5^b^	2.50 × 10^−15^
Number of capillaries per field	36.7 ± 8.2^a^	24.0 ± 7.2^b^	17.0 ± 5.3^c^	3.44 × 10^−4^
Number of fibres per capillary	6.3 ± 2.3^a^	2.3 ± 0.9^b^	2.9 ± 1.2^b^	1.20 × 10^−4^
Fibre surface per capillary, µm^2^	12961 ± 3985^a^	20169 ± 4248^b^	22150 ± 5681^b^	3.89 × 10^−3^
Fibre size, µm^**^	25.0 ± 1.8^a^	55.2 ± 8.5^b^	48.2 ± 11.4^b^	5.22 × 10^−6^
Coefficient of variation of fibre size, %	25.5 ± 2.0^a^	37.8 ± 7.9^a^	50.5 ± 10.7^b^	3.14 × 10^−3^

^*^Relative area (%) occupied by collagen VI or adipose tissue.

^**^Calculated from the Feret’s minimum diameter.

^a,b,c^Analysis of variance performed with the group factor for each trait. Different superscripts in each line indicate significant differences between groups, as determined by pairwise comparison (p-values ≤ 0.05).

Capillary density and fibre size were measured using laminin-fibronectin co-immunolabelling (Fig. 1D–F). The number of capillaries in the microscopic field was 1.5 and 2 times lower in severely affected muscles (FG-WSWB) than in macroscopically unaffected muscles (FG-C) (p-value ≤ 0.05) and normal muscles (SG) (p-value ≤ 0.0001), respectively. In addition, the structure and location of the capillaries appeared to be modified in FG-WSWB muscles as we noticed the presence of lumens in their capillaries, likely because of vasodilatation, and of connective tissue extensions that altered the connection between capillaries and muscle fibres (Fig. 1D–F).

As expected, the number of fibres in a microscopic field was significantly lower in FG than in SG muscles (p-values ≤ 0.0001) due to the difference in fibre size (measured as the minimal Feret’s diameter), which was significantly lower in SG than in FG muscles (p-values ≤ 0.0001) (Table 1). The number of fibres per capillary was approximately 2 to 3 for FG muscles and approximately 6 for SG muscles. However, if we consider the fibre surface supplied by one capillary, it was almost two times larger in FG (20169 µm² for FG-C and 22150 µm² for FG-WSWB) than in SG muscles (12961 µm²) (p-values ≤ 0.05) due to the difference in fibre size and conjunctive tissue extension. Interestingly, neither the number of fibres per capillary nor the averaged fibre size was different between FG-C and FG-WSWB muscles. However, there was significantly more fibre size variation in FG-WSWB muscles than in SG and FG-C muscles (p-values ≤ 0.01), probably due to the strong regeneration process indicated by an increase in frequency of regenerative fibre foci that contain more basophilic fibres with a great proportion of central nuclei.

### Differential gene expression analysis

To gain a better understanding of the molecular pathways underlying *pectoralis major* muscle susceptibility to WS and WB defects, a custom chicken 8 × 60 K Gene Expression Agilent Microarray was used to perform the transcriptomic analysis of muscles obtained from the SG, FG-C and FG-WSWB groups. Among the 61,657 probes spotted on the array, 43,688 (71%) were expressed, and 36,473 (59%) were annotated (corresponding to 12,272 genes) based on the NCBI EntrezGene database and Galgal5 chicken genome assembly. Based on quantitative histological characterization and a multidimensional scaling plot of gene expression, three samples (i.e., the two samples previously identified in the histological analysis as misclassified into groups according to the macroscopic examination of defects and another sample from the FG-C group) were considered outliers and were not kept in the differential analysis. The analysis of 21 samples was performed using a linear model and pairwise comparisons and revealed that 10,482 of the annotated genes were differentially expressed (DE) genes with a fold-change |FC| ≥ 1.2 in at least one of the three comparisons (adjusted p-value < 0.05). Among these, 6,725 genes were DE between SG and FG-C, 7,887 were DE between FG-C and FG-WSWB, and 9,608 were DE between SG and FG-WSWB. The log2-transformed FC of the DE genes ranged from −3.48 to 6.64 for FG-C *vs*. SG, from −6.81 to 9.19 for FG-WSWB *vs*. SG and from −3.87 to 4.22 for FG-WSWB *vs*. FG-C, indicating the presence of substantial gene expression differences between these groups.

The top 10 DE genes in the three comparisons based on adjusted p-value and log2 FC are presented in Table 2 and Supplementary Table S2, respectively. These genes were mainly involved in the extracellular matrix, collagen fibrils and cytoskeleton organization, muscle contraction, the inflammation response, calcium homeostasis, oxidoreductase activity and angiogenesis.

**Table 2:** Top 10 DE genes for each comparison based on adjusted p-value.

Gene symbol^*^	Gene ID	Description	Adjusted p-value	log FC
**FG.C vs. SG**				
**MUSTN1**	404773	musculoskeletal, embryonic nuclear protein 1	9.39 × 10^−14^	5.02
GOLGB1	426868	golgin B1	1.36 × 10^−12^	−2.80
**TNNT2**	396433	troponin T2, cardiac type	1.63 × 10^−12^	4.87
RGCC	418833	regulator of cell cycle	1.63 × 10^−12^	4.09
PIH1D3	422184	PIH1 domain containing 3	2.27 × 10^−12^	−2.25
MTPN	395702	myotrophin	3.82 × 10^−12^	1.83
DUSP5	423890	dual specificity phosphatase 5	4.12 × 10^−12^	3.84
SPP1	395210	secreted phosphoprotein 1	4.20 × 10^−12^	6.45
**RPS6KA1L**	396579	ribosomal protein S6 kinase A1-like	4.20 × 10^−12^	2.37
RUNX1	396152	runt-related transcription factor 1	7.43 × 10^−12^	3.22
**FG-WSWB vs. SG**				
**TNNT2**	396433	troponin T2, cardiac type	6.44 × 10^−16^	7.08
TNNT1	396009	troponin T1, slow skeletal type	2.09 × 10^−15^	5.85
TUBAL3	416694	tubulin alpha like 3	2.09 × 10^−15^	4.38
KCNK2	770954	potassium two pore domain channel subfamily K member 2	2.09 × 10^−15^	4.79
**RPS6KA1L**	396579	ribosomal protein S6 kinase A1-like	2.09 × 10^−15^	3.35
**MUSTN1**	404773	musculoskeletal, embryonic nuclear protein 1	2.09 × 10^−15^	5.18
APTX	395173	aprataxin	2.09 × 10^−15^	−2.86
MYH15	395534	myosin heavy chain 15	3.12 × 10^−15^	7.37
**MDK**	423196	midkine	4.40 × 10^−15^	5.21
VDAC1	416320	voltage dependent anion channel 1	4.40 × 10^−15^	−2.76
**FG-WSWB vs. FG-C**				
AKR1D1	418107	aldo-keto reductase family 1 member D1	1.91 × 10^−11^	4.08
FBLN1	373979	fibulin 1	5.16 × 10^−11^	3.85
FN1	396133	fibronectin 1	6.17 × 10^−11^	2.42
TNFAIP6	424315	TNF alpha induced protein 6	6.86 × 10^−11^	3.38
**MDK**	423196	midkine	7.21 × 10^−11^	3.62
ENPP2	420361	ectonucleotide pyrophosphatase/phosphodiesterase 2	1.72 × 10^−10^	2.93
OLFML3	419882	olfactomedin like 3	1.72 × 10^−10^	1.98
FKBP10	427013	FK506 binding protein 10	1.72 × 10^−10^	1.94
CCDC180	772014	coiled-coil domain containing 180	3.59 × 10^−10^	−1.76
CTHRC1	420262	collagen triple helix repeat containing 1	5.66 × 10^−10^	4.22

*Common genes between two or three comparisons are indicated in bold.

**Figure 2:**
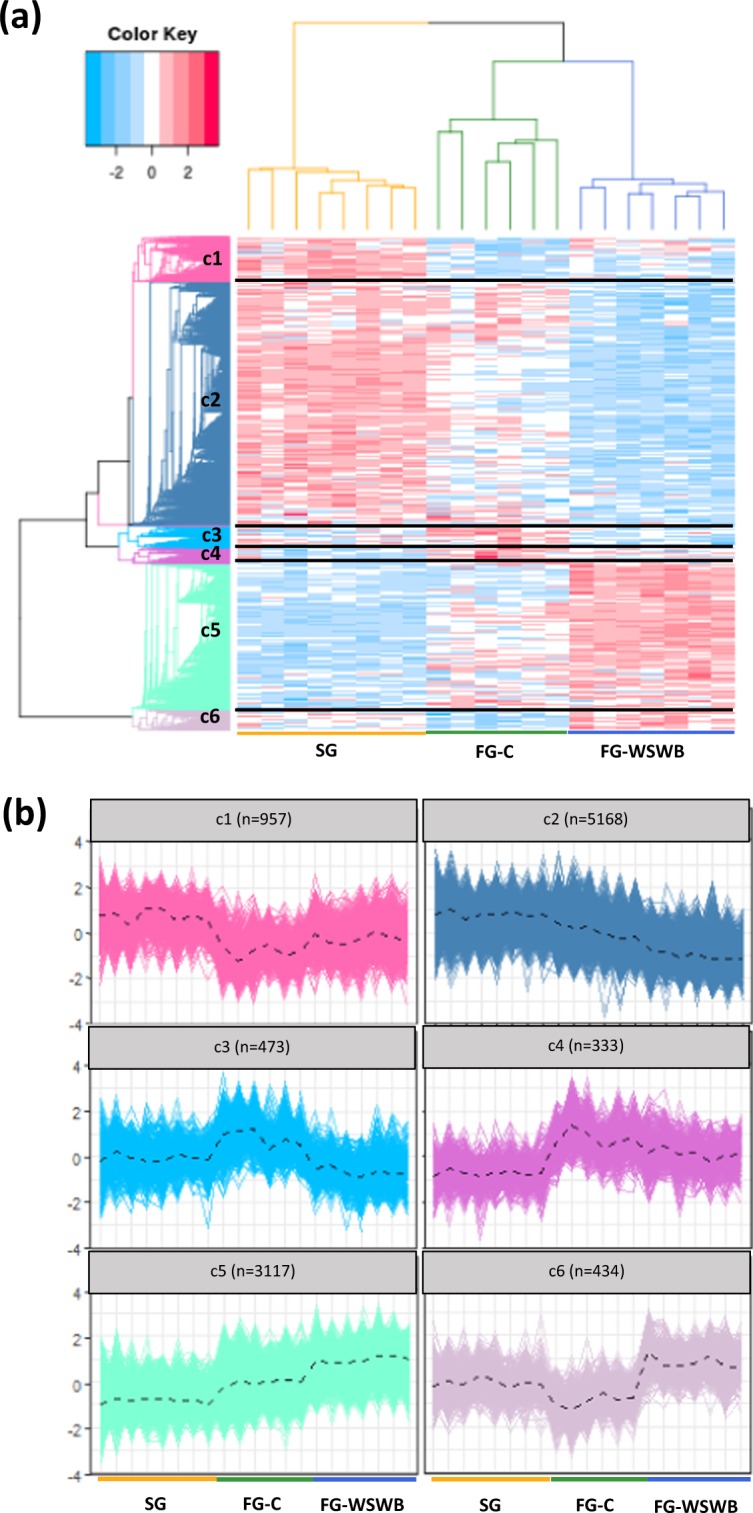
Heat map gene cluster classification of the SG, FG-C and FG-WSWB samples (**a**). Samples are shown in columns and DE genes in rows, and the scaled expression levels are depicted by a colour gradient: upregulated and downregulated genes are shown in red and blue, respectively. DE genes were grouped by a hierarchical clustering analysis in both rows and columns. Hierarchical clustering for the scaled gene expression matrix was based on the Pearson correlation distance and the average link aggregation distance. A modular height cut-off value in the hierarchical tree allowed the identification of six clusters (c1-c6) of DE genes with similar expression profiles (**b**). The dashed line indicates the average expression profile. The number of DE genes belonging to each cluster is indicated in parenthesis.

A hierarchical clustering was applied to the list of 10,482 DE genes with an |FC| ≥ 1.2 to group them into clusters according to their expression profile. A modular height cut-off value in the hierarchical tree allowed the identification of six clusters of DE genes that showed similar profiles of expression within clusters and a clear discrimination among the three groups (Fig. 2a). The number of genes and the expression profile of each cluster is presented in Fig. 2b. Clusters c1 and c4, which represented 12% of the DE genes, discriminated SG from FG muscles (regardless of the presence of defects). In all, 79% of DE genes were found in clusters c2 and c5, which were associated with both growth rate variations and the severity of defects. The expression of the 3,117 DE genes in cluster c5 gradually increase, while that of the 5,168 DE genes belonging to cluster c2 decreased from SG to FG-C and FG-C to FG-WSWB. Among all DE genes, only 9% appeared to be regulated between FG muscles free or affected by WS and WB regardless of the effect of growth. They were expressed at either higher levels (cluster c6) or lower levels (cluster c3) in the FG-WSWB group than in the FG-C and SG groups.

### Functional analysis of DE genes

All expressed genes on the array were annotated by Gene Ontology for Biological Process (BP) based on NCBI EntrezGene chicken IDs (taxonomic id: 9031) and orthologues. Among the 12,272 expressed genes on the array, 6,232 were annotated with at least one BP GO term (51%). The enrichment analysis was performed independently for each DE genes cluster with all expressed genes used as the background (Supplementary Data S1). Two hundred and sixty-seven GO terms were enriched in at least one cluster (p-value < 0.01), and 264 were unique, indicating that clusters of genes were very specific and different from each other (Table 3). The number of enriched GO terms in each cluster varied between 8 (cluster c3) and 128 (cluster c5).

**Table 3:** Functional enrichment of each cluster of DE genes.

Clusters	Number of DE genes	Number of enriched terms	Main enriched function
Cluster c1	957	24	metabolic process (protein, glycogen, nucleic acid); immune system (lymphocyte, immune effector process); signal transduction (intracellular steroid hormone receptor)
Cluster c2	5168	51	metabolic process (cellular respiration, aerobic respiration, protein, ATP, nucleic acid); gene expression; cellular component organization (vacuole, Golgi, autophagosome); macromolecular complex assembly (mitochondrial respiratory chain complex); actin cytoskeleton organization
Cluster c3	473	8	cellular process (apoptosis, cytokinesis); response to stimulus (oxidative stress, epidermal growth factor); regulation of transcription
Cluster c4	333	18	metabolic process (protein); response to stimulus (adaptive immune response, cellular response to tumour necrosis); signal transduction
Cluster c5	3117	128	metabolic process (protein, lipid, carbohydrate); biosynthetic process (collagen, lipid); organization (actin cytoskeleton, collagen fibril, extracellular matrix); body defence, immune system; cell migration, adhesion, proliferation (skeletal muscle satellite cell, fibroblast), development, morphogenesis (angiogenesis)
Cluster c6	434	38	cell migration, adhesion, proliferation; signal transduction (Wnt, Rho protein signalling pathway); transport (oxygen); actin cytoskeleton organization; metabolic process (protein, proteoglycan); neurogenesis

### Co-expression network analysis and module-trait relationships

The histological study identified fine phenotypes to accurately quantify the degree of muscle damage and significantly improve diagnosis, which is currently based on visual notation or muscle palpation after slaughter. Thus, studying the correlations between gene expression levels and histological quantitative phenotypes using gene network construction served as a complementary approach to the differential analysis. Moreover, this approach made it possible to make no a priori conclusions about the groups since it was based on continuous phenotypic variables and thus kept all the samples (n = 24) and took into account the variability in cell disorders highlighted at the histological level in the correlation analysis. From the normalized gene expression matrix, a weighted correlation network analysis (WGCNA) was performed to investigate the relationship between modules of co-expressed genes and histological traits and identify candidate biomarkers of the defects. Only the histological phenotypes allowed us to discriminate muscles severely affected by WS and WB from the two other groups based on, i.e., collagen VI and adipose tissue contents, the number of capillaries per field and the coefficient of variation of fibre size (Table 1) were kept for this analysis. Fifteen co-expression modules containing 50 to 6516 genes were constructed. In Supplementary Fig. S1, correlations between eigengenes and quantitative phenotypes show that several modules are related to one or more histological traits. We chose to focus on modules that had a mean gene significance (i.e., mean of correlations between gene expression and phenotype, GS) greater than 0.5 (Supplementary Fig. S2) and absolute value of the module-histological trait correlation higher than 0.7 (Supplementary Fig. S1). Hence, the light green (55 genes), royal blue (140 genes), brown (6516 genes), black (420 genes), and blue (3393 genes) modules were selected for further analysis. The light green module was strongly positively related to collagen VI content (0.89) and to variation in fibre size (0.85) but negatively related to the number of capillaries (−0.71). Brown and royal blue modules were negatively related to collagen VI content (−0.87 and −0.76, respectively) but strongly positively related to the number of capillaries (0.73 and 0.79, respectively). The blue module was negatively related to collagen VI content (−0.79) and variation in fibre size (−0.74), while the black module was positively correlated with variation in fibre size (0.84).

Genes for which expression was both highly correlated with a histological phenotype (GS ≥ 0.7) and significant modules eigengene (Module Membership, MM ≥ 0.7) were considered hub genes. Hence, 4,894 hub genes were associated with collagen VI labelling, 1,456 with the number of capillaries and 915 with variation in fibre size.

### Identification of candidate biomarkers for severe myopathies

**Figure 3:**
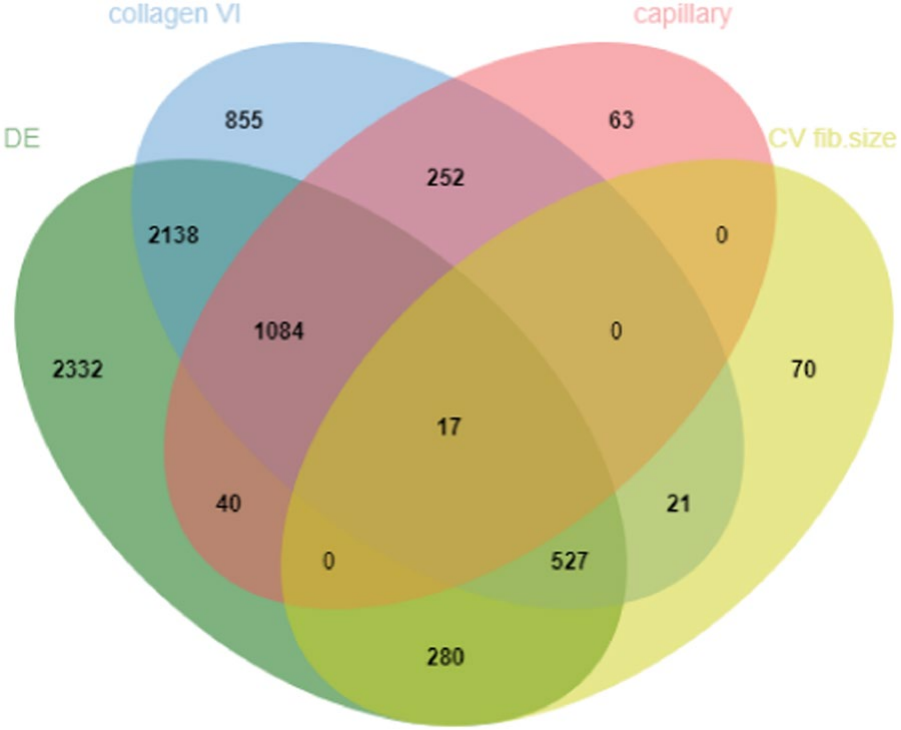
Venn diagram showing the number of genes that were common between two or more analyses (DE: gene differentially expressed (adjusted p-value <0.05) between FG-C and FG-WSWB muscles, collagen VI: hub genes for collagen VI labelling, capillary: hub genes for number of capillaries, CV fib. size: hub genes for variation in fibre size).

Genes that were DE between FG-C and FG-WSWB muscles (adjusted p-value < 0.05) and hub genes identified in the weighted correlation network analysis were combined in a Venn diagram to identify the most confident biomarkers related to muscle disorders involved in severe WS and WB pathologies (Fig. 3).

Among the 6,418 DE genes between FG-C and FG-WSWB muscles, more than one-third (36%) were not found to be strongly correlated with any of the three histological phenotypes studied. The vast majority of hub genes were DE between FG-WSWB and FG-C muscles. Only 17, 4, and 8% of hub genes that were related to collagen, number of capillaries, and variation of the fibre size, respectively, were not DE between FG-WSWB and FG-C muscles. Among the 7,679 genes included in the Venn diagram, 17 were common among all the lists, 1,611 (21%) between 3 lists, and 2,731 (36%) between two lists. Except for the 17 genes that were common to all the lists, no gene was common between the lists of genes correlated with the number of capillaries and variation in fibre size or between the lists of genes correlated with each of the three histological phenotypes. A total of 548 genes were common between the list of genes related to collagen VI and variation in fibre size, of which 527 were DE between FG-WSWB and FG-C muscles. Finally, 1,336 genes were highly correlated with both collagen VI and the number of capillaries, of which 1,084 were DE between FG-WSWB and FG-C muscles.

### Validation using RT-qPCR for a subset of genes

**Figure 4:**
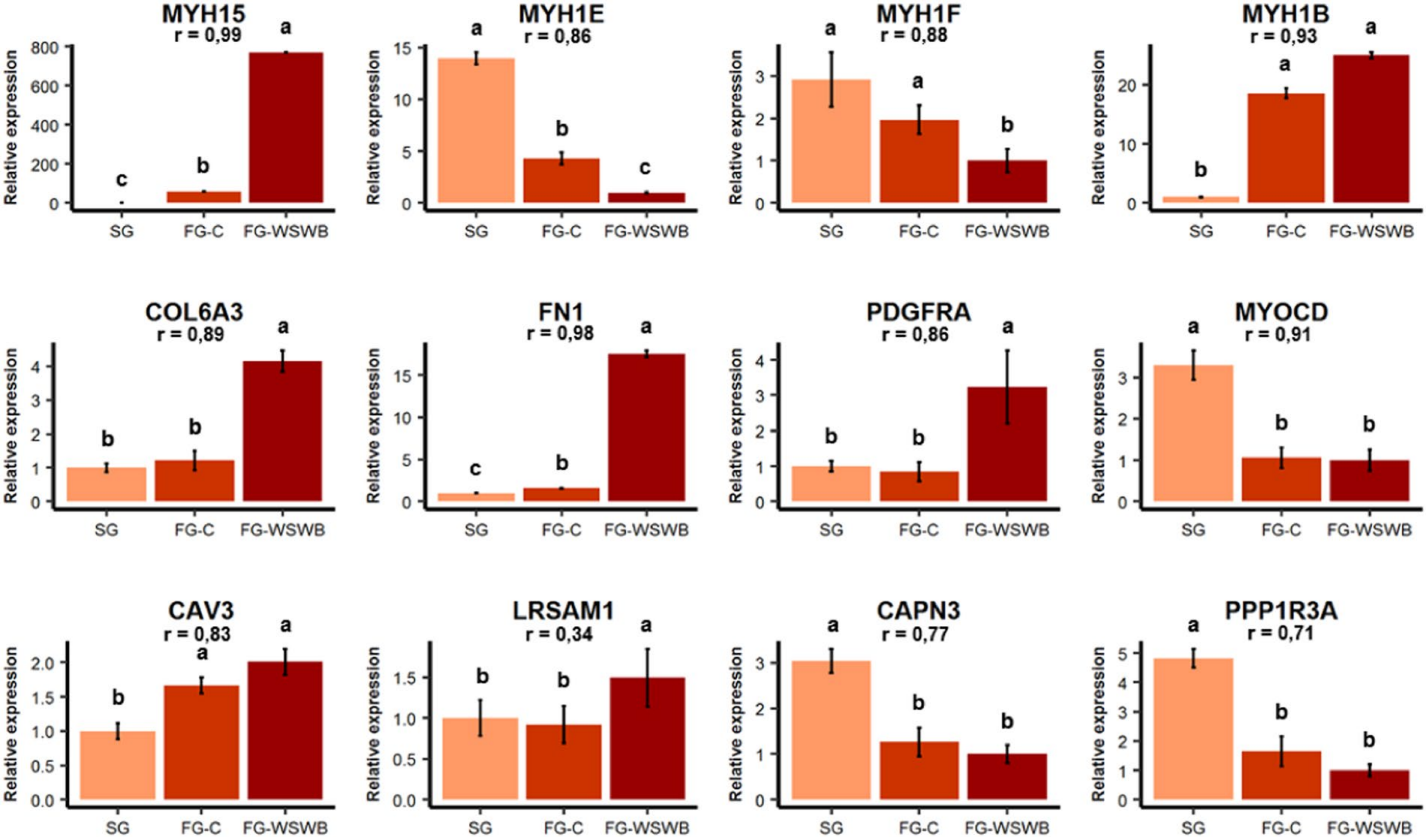
Relative expression by RT-qPCR of the 12 genes used for validation. The relative level of gene expression in each group was normalized according to the lowest value, which was arbitrarily defined as 1. Error bars indicate ± SE. ^a-b-c^Analysis of variance performed with the group factor for each gene. Different superscripts above bars indicate significant differences between groups, as determined by pairwise comparison (p-values ≤ 0.05). The r coefficient indicates the Pearson’s correlation between gene expression measured by RT-qPCR and microarray.

Among the genes found to be DE among the groups, a subset of 12 genes was chosen for validation by RT-qPCR. Genes were selected according to several criteria, including their functional role, their belonging to clusters, and, for some of them, their location within QTL regions recently identified for WS defect in broiler chickens^18^. The expression level of DE genes obtained by RT-qPCR was compared with expression obtained by microarrays. For each tested gene, the DE between groups observed on the microarray was confirmed with the exception of one gene, *LRSAM1*, the expression of which was only slightly correlated between RT-qPCR and microarray results (0.34). Except for this gene, expression measured by RT-qPCR and microarray was highly correlated, as shown by Pearson’s correlation coefficients between 0.71 and 0.99 (Fig. 4). Moreover, the gene expression values between groups obtained by qPCR were consistent with the categorization of these genes to different clusters.

Among the identified genes, the expression of the myosin heavy chain 15 (*MYH15*), which is involved in the contraction, development, and regeneration of avian skeletal muscles^24^, was dramatically higher (×771) in FG-WSWB than in SG muscles, while the difference between FG-C and SG muscles was much less dramatic (×60). As for *MYH15*, fibronectin 1 (*FN1*) expression was the highest in FG-WSWB and the lowest in SG, but the difference between the FG groups was much lower than that for *MYH15*. Platelet-derived growth factor receptor alpha (*PDGFRA*), collagen type VI alpha 3-chain (*COL6A3*) and leucine-rich repeat and sterile alpha motif-containing 1 (*LRSAM1*) were expressed at significantly highest levels in FG-WSWB than in FG-C and SG muscles. Conversely, the expression levels of adult (*MYH1E*) and neonatal (*MYH1F)* myosin heavy chain isoforms were lower in the FG-WSWB group than in the SG and FG-C groups. Other genes varied between the slow-growing and fast-growing groups, regardless of the presence of WS and WB defects. This was the case for caveolin 3 (*CAV3*) and embryonic myosin heavy chain isoform (*MYH1B*), whose expression was higher in FG muscles, and for myocardin (*MYOCD*), calpain 3 (*CAPN3*) and protein phosphatase-1 regulatory subunit 3 A (*PPP1R3A*), whose expression was lower in FG muscles than in SG muscles.

## Discussion

In recent years, the broiler industry has faced the emergence of serious meat quality defects, such as WS or WB, which affect both the competitiveness and the image of poultry production. Although research has intensified on this subject in recent years, the aetiology of WS and WB defects is still not understood except that it is now well-established that WS and WB conditions relate to an abnormal extent of connective and adipose tissues, which is associated with muscle fibre necrosis and regeneration processes^2,4^. One hypothesis is that these processes are the result of a long-term effect of genetic selection and intensification of production methods that have led to huge progress in terms of broiler growth and breast muscle development, which have also likely reached physiological limits. The comparison of muscles obtained from slow-growing chickens with muscles obtained from fast-growing broilers either affected or not affected by WS and WB defects revealed more than 10,000 DE genes. Part of the differential gene expression is due to change in the relative cell types present in the muscle, e.g. accumulation of adipocytes, fibroblasts and immune cells, and not strictly due to changes within myocytes. Our study revealed however many changes related to muscle fibre metabolism and contractile activity. Altogether, our observations allowed specifying structural and metabolic changes, which have progressively led to the onset of myopathies in fast-growing chicken (Supplementary Data S1).

### Long-term effects of selection on muscle structure and metabolism

Clusters c1 and c4 include genes whose expression in muscle has been altered either positively or negatively in response to long-term selection for growth (SG ≠ FG-C) but were not associated with the occurrence of WB and WS syndromes (FG-C = FG-WSWB). Their functions relate to non-specific biological processes and general functions, such as metabolic processes, signal transduction and the immune system (Table 3). Among these genes, those participating in the “positive regulation of glycogen metabolic process” (GO:0070875, Supplementary Data S1) function were expressed at lower levels in FG than in SG muscles, suggesting a decrease in the use of glycogen in fast-growing muscles independent of the presence of WS and WB defects. This finding is consistent with previous studies that clearly demonstrated the negative impact of selection for growth and/or breast meat yield on muscle glycogen content^25,26^. Among the genes likely to be involved in such changes, we identified *PPP1R3A*, whose expression was much lower in FG than in SG muscles (Fig. 4), as an important actor. *PPP1R3A* encode a muscle-specific regulatory subunit of protein phosphatase 1, which binds glycogen with high affinity, activates glycogen synthase, and inhibits glycogen phosphorylase. It has been reported that the overexpression of *PPP1R3A* leads to increased glycogen content^27^, while its KO results in a drastic reduction (90%) in the glycogen content in muscle^28^. Moreover, *PPP1R3A* was recently identified as the most interesting candidate gene for a major QTL region for ultimate pH (pHu)^29^ that reflects muscle glycogen reserves^30^. The depletion of glycogen reserves in the muscle of fast-growing lines could lead to a predisposing metabolic environment favourable to the development of myopathic syndromes. Indeed, many studies have reported that the occurrence of WS and WB defects is often associated with high meat ultimate pH^3,5,17,31–33^ or low muscle glycogen content^19,34^. Consistent with these findings, the occurrence and severity of WS were higher in a broiler line selected for high ultimate pH (pHu + ) than in its counterpart selected for low ultimate pH (pHu-)^17^. Moreover, in a recent genetic study, several QTL-controlling WS were identified in the pHu +  line but not in the pHu- one^18^, suggesting a possible interaction between the genetic control of WS and the metabolic status of muscle.

In addition to a decrease in glycogen stores, it has been shown that the haem pigment content of muscles also decreases with selection for growth and breast meat yields^25^, probably indicating that there is less muscle vasculature in fast than in slow-growing lines. In the present study, we observed that the expression of *MYOCD* was 3-fold lower in FG than in SG muscles. *MYOCD* is involved in extracellular matrix modelling and expressed in smooth muscle cell-containing tissues, in which it may play a crucial role in the differentiation of the smooth muscle cell lineage and vascularization. Interestingly, *MYOCD* was also recently identified in two different studies of QTL regions that control breast muscle yield^35^ and WS occurrence^18^ in slow- and fast-growing chicken lines, respectively. In parallel with the decrease in the expression of *MYOCD* observed in FG muscles (affected or not affected by defects), we report that compared to SG muscles, in FG muscles, there was a strong increase in the fibre surface supplied by one capillary (Table 1). Although this parameter was similar between FG-C and FG-WSWB chickens, we found that the extension of endomysial and perimysial connective tissues observed in the latter moved the capillaries away from the fibre membrane (Fig. 1D–F). This could be an aggravating factor that could further lower oxygen and nutrient supply to fibres in muscles affected by WS and WB defects.

Altogether, our observations formally demonstrate a recurrent assumption presented in the literature^2,19,26,36^, namely the suggestion that increasing breast muscle weight and yield via selection through hypertrophy of existing fibres has led to a lower capillary density. This may have led to an inadequate supply of oxygen and nutrients to the muscle fibres, which likely promotes the excessive use of glycogen and probably increases oxidative stress in fast-growing muscles.

In the present study, we also observed that *CAPN3* is expressed at 3-fold lower levels in FG than in SG muscles (Fig. 4). *CAPN3* is an intracellular protease specific to muscle that is involved in the calcium-dependent proteolytic system. A deficiency in this enzyme results in sarcomere disorganization and muscular limb-girdle muscular dystrophy type R1 (LGMDR1 calpain3-related) in humans^37^. In mice, its expression decreases following muscle denervation or denervation-devascularization injury, which induces muscle degeneration and regeneration^38^. Its lower expression in FG muscles is more consistent with the lower amount of vasculature observed in this line, as atrophy induced by denervation was not observed in such muscles. Although the embryonic myosin heavy chain isoform *MYH1B* is normally no longer expressed in adult muscle, its expression was increased in FG muscles (Fig. 4) and was one of the top 10 DE genes between the FG-C and SG groups (Supplementary Table S2). The expression of *CAV3*, which plays a key role in the fusion of myoblasts into myotubes during the maturation process of muscle fibres, was also higher in FG muscles than in SG muscles. These observations support the notion that selection for growth and breast muscle yield contributes to the occurrence of degeneration-regeneration processes in FG muscles, independent of the development of WS and WB myopathies.

### How has progressive metabolic modification due to selection resulted in myopathic disorders in broiler breast muscle tissues

If not directly related to myopathies, the metabolic and structural changes induced by long-term selection on growth performance and breast muscle yield and evidenced by functional analysis of clusters c1 and c4 have likely created a favourable physiological context for their onset. Clusters c2 and c5 indicate what types of biological processes are involved in the progression of myopathic defects in relation to growth variations. These two clusters accounted for almost 80% of the DE genes (Fig. 2).

Several genes belonging to cluster c5, in which gene expression progressively increased from SG to FG-C and from FG-C to FG-WSWB, are involved in the generation of superoxide anion (GO:0042554, Supplementary Data S1), a reactive oxygen species (ROS) issued from the first electronic reduction of oxygen during oxidative phosphorylation^39^. Among these genes, some contribute to the oxidative stress response (*ARNT*, *HIF1A*, *GPX8*, *ALDOC*, and *CRYAB*), and these include heat-shock proteins (*HSBP1*, *HSPA5*, *HSF2*, *HSPB7*, and *HSPH1*); these genes are known to be expressed at substantially higher levels during oxidative stress^40^. These results strongly support the notion that selection for growth progressively resulted in increased oxidative stress and that this process was exacerbated in fast-growing muscle affected by WS and WB. It is worth noting that elevated ROS levels contribute to human muscular dystrophies, such as Duchenne muscular dystrophy^41^, and that excessive ROS production alters calcium homeostasis via the release of calcium from the sarcoplasmic reticulum^19,36,42^ and leads to skeletal muscle necrosis and atrophy^43^. Indeed, several genes belonging to cluster c5 (such as *P2RX4*, *ATP2B1*, *ANXA6*, *ATP2A3*, *SLC8A1*, *ANXA2*, *ATP2A2*, and *SLC24A2*) participate in calcium ion transport, indicating the possible release of calcium at the origin of muscle calcium-dependent proteolysis and tissue degeneration^41^, which has been reported in WS and WB muscles.

Cluster c5 includes a great number of genes involved in the production of inflammatory molecules, such as cytokines, chemokines, cell adhesion molecules and tissue-degrading enzymes. They are grouped into several GO terms, including “inflammatory response” (GO:0006954), “positive regulation of cytokine secretion” (GO:0050715), “regulation of cytokine-mediated signalling pathway” (GO:0001959), “regulation of interleukin-1 secretion” (GO:0050704), “positive regulation of interleukin-12 production” (GO:0032735), and “positive regulation of tumour necrosis factor (TNF) production” (GO:0032760) (Supplementary Data S1). TNF is a multifunctional proinflammatory cytokine, and one of its isoforms (i.e., *TNFAIP6*) was in the top 10 DE genes between the FG-WSWB and FG-C groups (Table 2, Supplementary Table S2). It induces the expression of metalloproteinases, which are known to degrade several components of the extracellular matrix-cytoskeleton linkage in skeletal muscle and to thereby lead to extracellular alterations and myopathy^43^. Among the 14 matrix metalloproteinases included on the microarray, seven belong to cluster c5 (*MMP2*, *MMP10*, *MMP14*, *MMP16*, *MMP17*, *MMP24*, *MMP27*, and *MMP23B*). Consistent with the increase in the inflammatory response found in cluster c5, we observed the activation of the immune system cascade. This included 20% of the genes in cluster c5, with one of the three most significant functions the “innate immune response” (GO:0045087, Supplementary Data S1). We identified several chemokines, cell adhesion molecules and extracellular proteasomes that can favour the infiltration of macrophages, lymphocytes and neutrophils. Such infiltrations, observed in cases of muscle injury, are also frequently observed in muscles affected by WS and WB^1,2,4,14,44^. There are also several cytokines that are produced during the inflammatory response, such as *TNF-α* or *IL-6*, that play an important role in muscle regeneration. *TNF-α* attracts muscle satellite cells to the damaged site, promotes satellite cell proliferation by activating NF-κB signalling, and stimulates differentiation by activating the p38 signalling pathway. *IL-6* stimulates the migration, proliferation and differentiation of myoblasts^45^.

After injury, muscle regeneration mediated by muscle stem cells is switched on. Muscle stem cells (or satellite cells) are the major contributors to muscle regeneration and remain quiescent under normal conditions^45^. After muscle trauma, they are activated, giving rise to a proliferative myoblast population that differentiates to restore damaged myofibres. The “regulation of skeletal muscle satellite cell proliferation” (GO:0014842, Supplementary Data S1) was significantly enriched in cluster c5. It includes genes involved in the activation of muscle satellite cell proliferation (*FGF2*, *SDC4*, *GPC1*, *PAX7*, and *STAT3*), indicating the activation of the muscle repair process. This is consistent with the decrease observed in the expression of the adult and neonatal myosin heavy chain isoforms, *MYH1E* and *MYH1F*, which belong to cluster c2, and the increase observed in the expression of *MYH15*, which is involved in the regeneration of avian skeletal muscles^24^ and was one of the top 10 DE genes in the three comparisons (Supplementary Table S2). Moreover, cluster c5 also included several genes that contribute to collagen formation (GO:0032964, GO:0032967, GO:0030199), the GO term “collagen fibril organization” being one of the three most enriched terms of this cluster (Supplementary Data S1). All these observations were consistent with our histological study that showed that there was an increase in both the relative surface occupied by collagen VI across SG, FG-C and FG-WSWB muscles and the coefficient of variation of fibre size for FG-WSWB muscles compared to the other groups (Table 1). Collectively, our molecular and histological observations support the hypothesis that regeneration processes are more activated in fast than in slow-growing muscles, with this phenomenon exacerbated in muscles affected by WS and WB (Fig. 4).

Functional analysis of clusters c2 and c5 also revealed several enriched GO terms related to energetic pathways, especially glycogen metabolism, glucose use, cellular respiration and ATP production (Supplementary Data S1). This finding reveals that there was a progressive decrease (SG > FG-C> FG-WSWB) in the expression of genes encoding glycolytic enzymes (*PFKM*, *ALDOB*, *TPI1*, *GAPDH*, *PGK1*, *PGAM1*, *ENO3* and *PKM*), suggesting a lower level of anaerobic glycolytic activity, which is already observed in muscles affected by WS and WB^11,19,34,46^. This decrease in anaerobic glycolytic activity is concomitant with an alteration in mitochondrial cellular respiration. Indeed, several genes belonging to cluster c2 (SG> FG-C> FG-WSWB) were related to “cellular respiration” (GO:0045333), “aerobic respiration” (GO:0009060), “ATP synthesis coupled electron transport” (GO:0042773), and “mitochondrial respiratory chain complex I and IV assembly” (GO:0032981-GO:0033617) metabolic processes (Supplementary Data S1). These genes are primarily involved in the production of electron transport chain components in mitochondria (*CYCS*, *COX16*, *SCO1*, *SURF1*, *TIMM21*, *BCS1L*, *FOXRED2*…) and the Krebs cycle (*ACO2*, *PDHB*, *MDH1*, *MDH2*, *PDHA1*). Moreover, several genes belonging to cluster c5 (SG <FG-C <FG-WSWB) are involved in carbohydrate metabolic processes, such as gluconeogenesis (*FBP1*, *PCK2*, *GALE*, and *ALDOC*), carbohydrate derivative transport, nucleotide-sugar biosynthesis, the pentose phosphate pathway (*RBKS*, *RPE*, *TKT*, *ALDOC*, *DERA*, *FBP1*, *PGD*, and *PGM2*) and glycogenolysis (*GCG*, *INPP5K*). All these results strongly suggested that selection by itself has markedly modified the metabolic pathways used to produce energy in muscles and that these metabolic modifications are even more pronounced in muscles affected by WS and WB. This metabolic reorientation may be related to the progressive decrease in carbohydrate (especially glycogen) availability that has accompanied selection for growth and breast meat yield^25,26^ and that is also observed in muscles affected by WS or WB^5,21,34^. It is likely that these metabolic modifications have contributed to the appearance of muscle disorders in fast-growing birds. Indeed, it was recently shown that muscles exhibiting a heightened ability to store glycogen used carbohydrates as the main source of energy, whereas those lacking glycogen used energy produced from amino acid catabolism and lipid oxidation, leading to an adaptive response to oxidative stress and muscle regeneration processes^21,47^.

### Mechanisms of cellular defence and remodelling are specifically involved in case of severe myopathies

Functional analysis of clusters c3 and c6 highlighted the biological processes specifically activated in fast-growing muscles that are severely affected by WS and WB. Genes belonging to these clusters are regulated between FG-C and FG-WSWB regardless of the effect of growth. They account for 9% of the total DE genes and can be distinguished from that DE genes of clusters 2 and 5 (79% of DE genes) whose expression gradually changes in relation to growth variation and severity of defects. Cluster c6 contained genes that are overexpressed in cases of severe WS and WB. Its most enriched GO term is related to oxygen transport (GO:0015671, Supplementary Data S1) and encompasses genes encoding for oxygen carriers (*HBE*, *HBBA*, *HBM*, *HBA1*, and *HBBR*), likely indicating insufficient oxygen supply in muscles. Another group of functions relates to glycosaminoglycan and proteoglycan synthesis (“protein O-linked glycosylation via threonine” (GO:0018243) and “proteoglycan metabolic process” (GO:0006029)), which are both components of fibrous tissue (Supplementary Data S1). This is fully in agreement with histological observations that showed significant infiltration and extension of connective tissues within muscles (Fig. 1). At the molecular level, we noticed a strong increase in FG-WSWB muscles of that expressed *PDGFRA* (Fig. 4), a marker of fibro-adipogenic precursors (FAPs), which were recently characterized as playing a role in muscle regeneration and repair processes. Indeed, FAPs differentiate into fibroblasts and adipocytes under pathological conditions, such as muscular dystrophy, leading to connective and fat tissue infiltration^48^. Similarly, the expression of *COL6A3* and *FN1*, which respectively contribute to the formation of collagen VI and are found in most connective tissues, fibres and extracellular matrix (ECM) during tissue repair after degeneration, were expressed at substantially higher levels in FG-WSWB muscles (Fig. 4), in accordance with the high proportion of ECM that characterizes these muscles. *FN1* was also one of the top 10 DE genes between FG-WSWB and FG-C (Table 2).

Interestingly, cluster c6 was associated with several GO terms related to neurogenesis, such as “positive regulation of neurogenesis” (GO:0050769), “axonogenesis” (GO:0007409), “neuron differentiation” (GO:0030182), “positive regulation of synapse assembly” (GO:0051965), “regulation of neuron differentiation” (GO:0045664), and “neuron projection extension” (GO:1990138) (Supplementary Data S1). Genes belonging to these functions are mostly involved in axon guidance and the formation or maintenance of neuromuscular junctions (such as *AGRN*, *SLIT2*, *SEMA3A*, *EPHB3* and *NTRK2*). This is likely more a sign of the involvement of neuromuscular junction maintenance and repair than of motor neuron injury, which would result in foci containing atrophic fibres^49^, which were not observed in FG-WSWB muscles. Indeed, because neuromuscular junction structure alterations occur in affected muscles, axons would need to be attracted and redirected to neuromuscular junctions. Many factors that promote myofibre regeneration and repair, such as semaphorin 3A (*SEMA3A*), which is expressed by satellite cells in injured muscle, also serve as promoters of axonal sprouting and guidance and neuromuscular junction repair as nerves grow towards newly regenerated myofibres^50^. If cluster c6 is associated with the activation of several mechanisms, such as oxygen transport, axon guidance, and neuromuscular junction repair, then in the case of severe myopathy, cluster c3 could be associated with limitations in the development of cellular defence systems. Indeed, it contains genes whose expression is downregulated in muscles affected by WS and WB, with some of these genes participating in apoptosis and cytokinesis, two processes involved in cell defence or survival. This could be a sign that these cells fail to develop effective mechanisms of regeneration, and this defect is probably responsible for the substantial alteration of the structure of muscle and muscle functions observed in muscles severely affected by WS and WB.

### Identification of quantitative histological phenotypes and molecular markers for the diagnosis of muscle disorders in chicken

Our study, like other recent studies^19,20,22,35^, helps to advance our understanding of the biological processes that are involved in the development of myopathies in the breast muscle tissues of modern broiler lines. It also provides several finely defined histological phenotypes, which were used to precisely quantify the degree of muscle damage and significantly improve diagnosis, which is currently based on visual or palpation scoring of muscle after slaughter. The study of these fine histological phenotypes helps researchers to better describe the major structural changes that occur in muscles affected by WS and WB, and we also used them to determine, through a combination of DE analysis and weighted correlation network analysis, the most confident molecular markers related to muscle disorders involved in severe WS and WB pathologies (Fig. 3). Contrary to connective tissue content, fibre size variation and capillary density, no WGCNA module was found to be highly correlated with adipose tissue content. This is probably due to the great variability observed between samples from a same category (see standard deviations in Table 1). It is also possible that changes in fat metabolism could occur earlier during development, as recently shown in Papah *et al*.^23^, and that its later extension could interfere with that of connective tissue.

Our integrative analysis led to a set of 17 relevant biomarkers that were DE between the FG-C and FG-WSWB groups and highly correlated with histological phenotypes related to myopathy (Table 4). Three of these genes have not yet been characterized. Among the others, most are involved in muscle regeneration phenomena (*PDLIM1*), fibrosis (*COL6A2*), hypoxia (*ARNT2*, *HIF1AN*, and *PTGS1*) and axon guidance (*CADM1*, *SLIT3* and *NAV3*). These functions are the main biological processes that are specifically activated in fast-growing muscles severely affected by WS and WB defects (see the functional annotation of clusters c3 and c6). We also propose to enrich this set of biomarkers by 5 additional genes (*MYH15*, *FN1*, *COL6A3*, *MYH1E*, and *MYH1F*) that were strongly DE between the FG-C and FG-WSWB groups, were highly correlated to two of the three histological phenotypes studied, and were located in the QTL region controlling the WS defect in the breast muscle tissues of chicken^18^ (Table 4). Among these five genes, *MYH15* was the only one that was highly correlated with the number of capillaries. This slow-type myosin, also called ventricular myosin in chicken because it is mainly expressed in heart, is transiently expressed in embryonic chick skeletal muscles and then re-expressed during muscle regeneration^24^. Interestingly, it is associated with bovine pulmonary hypertension related to high altitude, suggesting that *MYH15* could be involved in the response to hypoxic conditions^51^. The ability of this set of 22 genes to discriminate muscles with severe myopathies from non-affected muscles should now be tested in larger populations obtained from different genetic backgrounds. These biomarkers based on gene expression provide fine diagnostic tools to quantify WS and WB defects. Although they require invasive muscle sampling, they can serve as intermediate phenotypes for the search of non-invasive predictive measurements, based on blood test or imaging on live animal for instance.

**Table 4:** Set of candidate biomarkers of severe breast muscle myopathies identified based on combinations of genes that were differentially expressed between FG-WSWB and FG-C muscles and the WGCNA analyses as well as QTL mapping for WS.

Gene Symbol	Gene ID	Description
ARNT2	415481	aryl hydrocarbon receptor nuclear translocator 2
EYA2	395745	EYA transcriptional coactivator and phosphatase 2
PTGS1	427752	prostaglandin-endoperoxide synthase 1 (prostaglandin G/H synthase and cyclooxygenase)
CADM1	419762	cell adhesion molecule 1
SLIT3	374173	slit guidance ligand 3
PDLIM1	428948	PDZ and LIM domain 1
GPR162	418288	G protein-coupled receptor 162
TBC1D19	422800	TBC1 domain family member 19
COL6A2	396292	collagen type VI alpha 2 chain
HIF1AN	428952	hypoxia inducible factor 1 subunit alpha inhibitor
LRWD1	417511	leucine rich repeats and WD repeat domain containing 1
NAV3	417869	neuron navigator 3
LOC421935	421935	uncharacterized
ADORA1	374212	adenosine A1 receptor
LOC107051073	107051073	uncharacterized
ATP8B1	426857	ATPase phospholipid transporting 8B1
C9H21ORF2	424866	chromosome 9 C21orf2 homolog
***Located in a QTL region for WS***		
MYH15	395534	myosin heavy chain 15
COL6A3	396548	collagen type VI alpha 3 chain
FN1	396133	fibronectin 1
MYH1E	427788	myosin heavy chain 1E, skeletal muscle
MYH1F	768566	myosin, heavy chain 1F, skeletal muscle

In conclusion, the present study provides new information on the biological processes that lead to the occurrence of severe myopathy in the breast muscles of modern broiler lines. By comparing the muscle transcriptome of slow-growing to fast-growing broilers that were affected or not affected by myopathies, we attempt to specify molecular pathways that were altered by the long-term selection of growth performance to those directly involved in the development of muscle disorders. Our data suggest that selection for fast growing and breast meat yield has progressively led to inadequate supplies of oxygen and the nutrients that promote carbohydrate depletion in muscle. Such changes would have provided favourable conditions for metabolic modifications that shift cells toward use of alternative catabolic pathways to produce energy, leading to an adaptive response to oxidative stress and the first signs of inflammatory, regenerative and fibrotic processes. All these processes appeared to be dramatically intensified in muscles affected by myopathies, as are new mechanisms related to cellular defence and remodelling to allow the muscles to function (oxygen transport, neurogenesis). Finally, our study highlights the fine histological phenotypes and genes that have expression profile that are highly correlated with muscle disorders involved in the development of myopathies, thus opening new perspectives for a more fine diagnosis and a better screening tool for broilers affected these such defects.

## Methods

### Animals, sample collection, muscle and meat quality measurements

The present study was carried out on *pectoralis major* muscles obtained from a fast- and a slow-growing chicken genotype. The experimental slow-growing (SG) chicken line was created by INRA in the seventies’ from three original strains: Bresse-Blanche, New-Hampshire and White-American breeds. Although this line has been selected for improved body weight, its growth rate is close to that of chicken strains currently used for the French “Label Rouge” (free-range) production^52^. The fast-growing (FG) line is a grand-parental commercial modern line highly selected by Hubbard for growth and breast muscle yield. Birds of the two genotypes were reared following the same standard practices. They were raised separately but received the same diet. All animals were slaughtered at 6 weeks of age at PEAT INRA Poultry Experimental Facility (2018, 10.15454/1.5572326250887292E12). The slaughter and carcass processing conditions of the FG broilers are fully described in Alnahhas *et al*.^53^. Samples of the *pectoralis major* muscle were collected fifteen minutes after slaughter and either directly snap-frozen in liquid nitrogen for molecular analysis or stored in isopentane cooled with liquid nitrogen for histological purposes. All muscle samples were then stored at −80 °C until further analysis. One day after slaughter, three trained people visually graded *pectoralis major* muscles for WS and WB. Both WS and WB were categorized separately on a three-point scale: 0 = absence of defects, 1 = moderate severity of defects, and 2 = high severity of defects. Defects were scored according to the widths of white stripes based on the scale of Kuttappan *et al*.^6^ for WS in addition to visual characteristics and hardness on palpation for WB.

Body composition was characterized for all SG and FG birds through the measurement of *pectoralis major* yield (PMY) expressed in relation to body weight (BW). Breast meat yield (BMY), abdominal fat yield (AFP), and *pectoralis minor* yield (PmY) were also measured in FG birds as were several breast meat quality traits that were evaluated in *pectoralis major* muscle: ultimate pH (pHu) and the colour parameters L*, a*, b*, which were measured 24 hours post-mortem, drip loss (DL) during a 4-day storage period, cooking loss (CL), Warner Bratzler shear force (SF) of cooked meat, curing-cooking yield (CCY) and the thiobarbituric acid-reactive substance index (TBA-RS), used as markers of lipid peroxidation and measured after 8 days of storage. All these measurements were obtained as described in Alnahhas *et al*.^17^.

Three groups of eight animals were selected for further molecular and histological analyses: eight animals obtained from the SG line and two groups of eight animals selected from among the 200 FG birds according to their extreme visual score for WS and WB defects and DL, CL and CCY phenotypes. The 8 FG-C birds were visually free of any defects. They were selected within their group based on their high water retention capacity (with below-average DL and CL and above-average CCY). The 8 FG-WSWB birds were affected by both the WS and WB defects (average score of 2 for both WS and WB) and have a CCY below the average of their group.

### Histochemical traits

Immunohistochemistry was performed on 10 µm-thick *pectoralis major* muscle cross-sections obtained from the FG-C, FG-WSWB and SG groups. All primary antibodies were obtained from the Developmental Studies Hybridoma Bank, created by the NICHD of the NIH, maintained at The University of Iowa, Department of Biology, Iowa City, IA, and developed by Douglas M. Fambrough, as follows: 39 (anti-collagen VI), B3/D6 (anti-fibronectin), and 31/31-2 (anti-laminin). Secondary antibodies and conjugated streptavidin were obtained from Southern Biotech, Birmingham, AL, USA, as follows: goat anti-mouse IgG1 conjugated to a TR antibody, goat anti-mouse IgG H+L conjugated to a biotin antibody, and streptavidin conjugated to Cy2. The washing procedure consisted of incubating cross-sections for 3 × 5 minutes in phosphate-buffered saline solution. Slides were mounted in Mowiol medium (Sigma, Lyon, France) and stored at 4 °C in a dark chamber before they were observed. Whatever the labelling procedure, six images per sample were selected randomly (captured at 10× and/or 20× magnification using a Leica MC170 camera; Leica Microsystems, Nanterre, France) on a Leica DMRB microscope.

#### Fibrosis and adiposis quantification

The Collagen VI-Bodipy co-labelling procedure was used to quantify the extension of connective and adipose tissues within muscle. Muscle cross-sections were fixed with 4% paraformaldehyde solution, incubated in 10% goat serum (Sigma, Lyon, France) for 30 minutes, and then incubated with primary Collagen VI antibody (1/200) for 2 hours. After the tissues were washed, muscle cross-sections were incubated with a secondary goat anti-mouse IgG1 conjugated to a TR antibody (1/500) for 1 hour, washed again, and incubated with Bodipy 493 (4,4-Difluoro-1,3,5,7,8-Pentamethyl-4-Bora-3a,4a-Diaza-s-Indacene, 1/3000, Sigma, Lyon, France) for 15 minutes. The relative surface areas labelled with collagen VI and Bodipy were quantified using the Area Fraction Parameter function in ImageJ software after transformation into black and white images (8-bit) and threshold adjustment.

#### Capillary density and fibre size measurement

A fibronectin-laminin co-labelling procedure was used to label capillary membranes (fibronectin) and basal lamina surrounding muscle fibres (laminin). Muscle cross-sections were incubated in 10% goat serum for 30 minutes and with primary fibronectin antibody (1/100) for 1 hour. After they were washed, cross-sections were incubated with secondary goat anti-mouse IgG H+L conjugated to biotin antibody (1/1000) for 45 minutes, washed, and incubated with streptavidin conjugated to Cy2 (1/500) for 45 minutes. After they were washed, the cross sections were incubated with primary laminin antibody (1/50) for 1 hour, washed, and finally incubated with secondary goat anti-mouse IgG1 conjugated to a TR antibody (1/1000) for 45 minutes. Capillaries were counted manually, and fibre size was determined using the minimum Feret’s diameter parameter function of the ImageJ software after transformation into black and white images (8-bit) and threshold adjustment.

### Microarray expression profiling

#### RNA extraction

Total RNA was extracted from *pectoralis major* muscle samples using the RNeasy® Mini kit (Qiagen, Valencia, CA, USA). Residual genomic DNA was removed by DNase I treatment (Qiagen, Valencia, CA, USA). RNA concentration was measured using a NanoDrop ND-1000 spectrophotometer (Thermo Fisher Scientific, Waltham, MA, USA), and RNA integrity was checked according to an RNA Integrity Number (RIN > 8) using RNA 6000 Nano chips run on a Bioanalyzer 2100 (Agilent Technologies, Santa Clara, CA, USA).

#### Labelling and hybridization

Muscle transcriptional profiling was performed using an 8 × 60 K Agilent *Gallus gallus* custom-commercial array (Agilent Technologies, Santa Clara, CA, USA) containing 61,657 probes (Platform GPL20588 in the U.S. National Center for Biotechnology Information Gene Expression Omnibus (GEO) microarray database). RNA labelling and microarray processing were performed by the @BRIDGe platform (INRA, UMR GABI, Jouy-en-Josas) using the procedure described in Jacquier *et al*.^54^. The microarray datasets were submitted to the GEO microarray database with the accession number GSE127806.

#### Re-annotation of the microarray chip on the current Galgal5 chicken genome

To compare the DE genes identified in this study and the genetic markers of WS provided by our previous study^18^, we re-annotated the chip.

The transcriptome alignment on the Galgal5 chicken assembly (EntrezGene database) of all the probes deposited on the chip was carried out using the *blastn* algorithm (hybridization tolerance of two mismatches and a single probe-associated gene as parameters), which is available in the BLAST + suite (ncbi-blast-2.6.0+)^55^, on the cluster obtained from the Genotoul bioinfo platform (http://bioinfo.genotoul.fr/). Probes were annotated on a Galgal5 assembly as reported in Supplementary Data S2.

Probes with a hybridization tolerance of two mismatches and a single probe-associated gene were considered correctly annotated and were kept for further analysis.

#### Differential expression analysis

First-step filtering was applied to keep only those probes that were expressed in this study (43,688 probes). The fluorescence signal for each probe was log2-transformed and normalized by median centring for each array. The differences in expression among the three groups (SG, FG-C, and FG-WSWB) were computed using the log2 transformation of the fold-change (log2FC). The differential analysis was performed using a linear model to identify DE probes among all pairwise comparisons of the three groups implemented in R package limma version 3.32.7 (Linear Model for Microarray Data)^56^. The p-values were adjusted for multiple testing by the Benjamini-Hochberg method^57^ to control the False Discovery Rate (FDR). Probes were considered DE when the adjusted p-value was ≤ 0.05 and |FC| ≥ 1.2.

#### Hierarchical clustering

To identify clusters of DE genes, hierarchical clustering was performed on the gene expression matrix. The correspondence between expressed probes and annotated genes was determined using the re-annotation of the chip. Genes with at least one Galgal5 annotated DE probe, |FC| ≥ 1.2 and the highest mean expression probe level in one of groups were kept. Normalized expression (after log2-transformation) was scaled for each gene. Hierarchical clustering on the scaled gene expression matrix was based on the Pearson correlation distance and an average link aggregation distance. A modular height cut-off value in the hierarchical tree obtained using the cutreeDynamic function of dynamicTreeCut package^58^ (with the following parameters: deepSplit = 1, minClusterSize = 300) was applied to identify clusters of DE genes that shared similar expression profiles across the three groups (FG-C, FG-WSWB and SG).

### Functional enrichment analysis

All the genes expressed on the chip were annotated by Gene Ontology^59,60^ for Biological Process categories according to the NCBI EntrezGene database using orthologues. Functional enrichment tests were performed using the ViSEAGO R package, which is available at https://forgemia.inra.fr/umr-boa/viseago. Enrichment for functions within each cluster of genes was tested using a Fisher’s exact test and the “elim” algorithm (p < 0.01) with all expressed genes used as the background.

### Co-expression network analysis

To study the correlations between gene expression levels and histological quantitative phenotypes as a complementary approach to the differential analysis, we applied a co-expression analysis using the R package Weighted Correlation Network Analysis (WGCNA)^61^.

#### Network construction

The co-expression analysis was performed using an expression matrix based on the 24 samples and 11,999 expressed and annotated genes. The unsigned connected network was built based on the adjacency matrix between genes. From the gene expression matrix, Pearson’s correlations between every pair of genes were computed and raised to a selected power of β = 7 using the pickSoftThreshold function to reach a scale-free topology index (R²) of at least 0.60. The adjacency matrix was turned into a Topological Overlap Measure (TOM) matrix, which can be used to assess the degree of shared neighbours between pairs of genes.

#### Module detection

A hierarchical clustering of the genes based on the TOM dissimilarity measure followed by a modular height cut-off value of branches in the hierarchical tree using the cutreeDynamic function (deepSplit = 4, minClusterSize = 30) was performed to detect modules of co-expressed genes. The module eigengene, which was the first principal component of each module and represented the expression value of each module, was calculated. Modules with expression profiles that were very similar (correlation = 0.90) were merged since there was a high probability that genes belonging to these modules are highly co-expressed.

#### Module-trait relationships

Only the histological phenotypes that allowed discrimination of muscles severely affected by WS and WB from other muscles (i.e., collagen VI labelling, adipose tissue, number of capillaries and coefficient of variation of fibre size) were kept for the analysis. The module eigengene was used to detect biologically relevant modules. Indeed, module-trait relationships were estimated using Pearson’s correlation between the module eigengene and the trait of interest.

Genes for each module with high Gene Significance (GS ≥ 0.7) corresponding to the absolute value of the correlation between gene expression and the histological trait and high Module Membership (MM ≥ 0.7), which was defined as the absolute value of the correlation of the module eigengene and the gene expression profile, were defined as hub genes.

### Validation by RT-qPCR

To validate the results obtained on the microarray, RT-qPCR was performed to examine the relative expression of 12 genes selected according to several criteria, including their functional role, their belonging to each of the identified clusters, and, for some of them, their location within QTL regions recently identified as controlling the WS defect in broiler chickens^18^. Total RNA was extracted from *pectoralis major* muscle samples using RNA NOW (Ozyme, St Quentin en Yvelines, France). Ten micrograms of RNA from each sample were reverse-transcribed using RNase H^−^MMLV reverse transcriptase (Superscript II, Invitrogen, Illkirch, France) and random primers (Promega, Charbonnières les Bains, France). Primers targeting the studied genes were designed with Primer3 version 4.0.0^62,63^. The list of primer sequences is available in Supplementary Table S3. The products of amplification were analysed by electrophoresis and further sequenced.

Gene expression was quantified by qPCR using a Roche LightCycler® 480 II (Roche Applied Science) and Takyon® mix (Eurogentec, Seraing, Belgium) according to the manufacturer’s recommendations. Quantitative PCR conditions were set at 95 °C for 5 min, followed by forty-five cycles of 10 s at 95 °C, 20 s at 60 °C and 10 s at 72 °C. *18S ribosomal RNA* was used as a housekeeping gene to normalize the CT values as its expression was invariant between groups. A mix of chicken p*ectoralis major* muscle cDNA served as a reference sample (control cDNA). The calculation of absolute mRNA levels was based on the PCR efficiency and the threshold cycle (CT) deviation of an unknown cDNA versus the control cDNA according to the equation proposed by Pfaffl^64^.

### Ethics approval

Experimentation was conducted in PEAT INRA Poultry Experimental Facility (2018, 10.15454/1.5572326250887292E12) and authorized for animal experimentation by the French veterinary service, which is the competent authority (D-37-175-1). The scientist in charge of the experimentation received training and personal authorization (37-112). In agreement with the Ethical Committee for Animal Experimentation in Val de Loire (C2EA-19), the experiments reported here did not require approval from a specific ethical committee since only classical rearing practices for broilers were used. No permissions were required to collect the chicken lines owned by INRA.

## Supplementary information

Supplementary files

Supplementary Data S1

Supplementary Data S2

## Data Availability

The microarray data were submitted to the Gene Expression Omnibus (GEO) microarray database (accession number GSE127806).
