# Supplementary material for: Differential expression and co-expression gene network analyses reveal molecular mechanisms and candidate biomarkers involved in breast muscle myopathies in chicken
Source: Sci Rep. 2019 Oct 17;9:14905. doi: 10.1038/s41598-019-51521-1 (PMC6797748; doi:10.1038/s41598-019-51521-1)
Supplement: Supplementary file 1 — Supplementary files [file 41598_2019_51521_MOESM1_ESM.pdf]

Differential expression and co-expression gene network analyses reveal molecular mechanisms and candidate biomarkers involved in breast muscle myopathies in chicken

Eva Pampouille[1,2], Christelle Hennequet-Antier[1], Christophe Praud[1], Amélie Juanchich[1], Aurélien Brionne[1], Estelle Godet[1], Thierry Bordeau[1], Maxime Banville[2], Elisabeth Le Bihan-Duval[1], Cécile Berri[1*]

[1]BOA, INRA, Université de Tours, 37380 Nouzilly, France

[2]Hubbard SAS, Mauguérand, 22800 Le Foeil - Quintin, France

[*]Corresponding author: cecile.berri@inra.fr

Supplementary Table S1. Means and standard deviations for body weight, body composition and meat quality traits measured at 6 weeks of age.

**Traits***	**FG-C (N=8)**	**FG-WSWB (N=8)**	**p-value****
**Growth and body composition**			
BW (g)	3213 ± 211	3435 ± 284	0.10
PMY (%)	17.06 ± 1.38	18.72 ± 1.14	**0.02**
PmY (%)	3.96 ± 0.30	3.74 ± 0.36	0.20
BMY(%)	21.0 ± 1.55	22.5 ± 1.41	0.07
AFP (%)	1.61 ± 0.27	1.79 ± 0.34	0.28
***Pectoralis major* quality traits**			
pHu	5.92 ± 0.11	5.91 ± 0.20	0.83
L*	50.1 ± 2.66	53.0 ± 2.95	0.07
a*	-0.93 ± 0.43	-0.92 ± 0.39	0.95
b*	10.2 ± 0.65	11.1 ± 1.35	0.14
DL (%)	2.09 ± 0.84	4.00 ± 0.95	**8.07×10^{-4}**
CL (%)	13.3 ± 2.95	19.2 ± 2.91	**1.26×10^{-3}**
CCY (%)	82.6 ± 2.45	78.2 ± 5.59	0.07
SF (N/cm^2)	12.8 ± 0.94	13.2 ± 1.63	0.56
TBA-RS	0.50 ± 0.16	0.59 ± 0.22	0.37

BW* body weight, *PMY pectoralis major* yield, *PmY pectoralis minor* yield, *BMY* breast meat yield, *AFP* abdominal fat percentage, *pHu pectoralis major* ultimate pH, *L lightness, *a** redness, *b** yellowness, *DL* drip loss, *CL* cooking losses, *CCY* curing-cooking yield, *SF* shear force value of the cooked meat, *TBA-RS* thiobarbituric acid-reactive substance of the raw meat.

**Two-tailed Welch's mean values equality t-test at 95% confidence level

Supplementary Table S2. Top 10 DE genes for each comparison based on log FC.

Gene symbol*	Gene ID	Description	log FC	Adjusted p-value
**FG.C vs. SG**				
**SPP1**	395210	secreted phosphoprotein 1	6.64	5.53×10^{-12}
**MYH1B**	374069	myosin heavy chain 1B, skeletal muscle (embryonic isoform)	5.97	2.19×10^{-11}
**CSRP3**	422979	cysteine and glycine rich protein 3	5.46	1.20×10^{-9}
MYH1A	417309	myosin heavy chain 1A, skeletal muscle	5.20	5.02×10^{-9}
**TPM3**	770103	tropomyosin 3	5.19	1.66×10^{-10}
MUSTN1	404773	musculoskeletal, embryonic nuclear protein 1	5.07	6.83×10^{-13}
**TNNT2**	396433	troponin T2, cardiac type	4.93	3.17×10^{-12}
**MYH15**	395534	myosin heavy chain 15	4.91	4.19×10^{-10}
LOC769899	769899	avidin-related protein 4/5-like	4.81	6.04×10^{-7}
PLN	396378	phospholamban	4.78	1.61×10^{-10}
**FG-WSWB vs. SG**				
**MYH15**	395534	myosin heavy chain 15	7.88	1.33×10^{-14}
**SPP1**	395210	secreted phosphoprotein 1	7.86	1.84×10^{-14}
**TPM3**	770103	tropomyosin 3	7.55	1.88×10^{-14}
**CSRP3**	422979	cysteine and glycine rich protein 3	7.36	6.54×10^{-13}
**PTX3**	548626	pentraxin 3	7.24	2.24×10^{-14}
TNNI1	421161	troponin I type 1 (skeletal, slow)	7.18	4.49×10^{-15}
**TNNT2**	396433	troponin T2, cardiac type	7.17	7.64×10^{-16}
OCM2	396531	oncomodulin 2	7.13	1.55×10^{-12}
**MYH1B**	374069	myosin heavy chain 1B, skeletal muscle (embryonic isoform)	7.12	1.71×10^{-13}
MYOZ2	422682	myozenin 2	6.75	3.21×10^{-14}
**FG-WSWB vs. FG-C**				
CTHRC1	420262	collagen triple helix repeat containing 1	4.22	5.66×10^{-10}
AKR1D1	418107	aldo-keto reductase family 1 member D1	4.08	1.91×10^{-11}
TNFAIP6	424315	TNF alpha induced protein 6	3.92	1.72×10^{-10}
FBLN1	373979	fibulin 1	3.85	5.16×10^{-11}
MDK	423196	midkine	3.84	6.18×10^{-10}
THBS2	414837	thrombospondin 2	3.77	6.30×10^{-10}
PTN	418125	pleiotrophin	3.53	1.18×10^{-6}
**PTX3**	548626	pentraxin 3	3.53	1.90×10^{-8}
FRMD5	396204	nephroblastoma overexpressed	-3.46	2.75×10^{-9}
**MYH15**	395534	myosin heavy chain 15	3.28	1.60×10^{-8}

*Common genes between two or three comparisons are indicated in bold.

Supplementary Data S1. Functional enrichment analysis of each cluster of DE genes that shared a similar expression profile (see Excel file Supplementary_Data_S1.xlsx).

Supplementary Figure S1. Relationships between gene expression modules and histological traits. Each row corresponds to a module eigengene, and each column corresponds to one histological trait. Each cell contains the corresponding correlation and its associated p-value (shown in brackets). The table is colour-coded by correlation according to the colour legend.

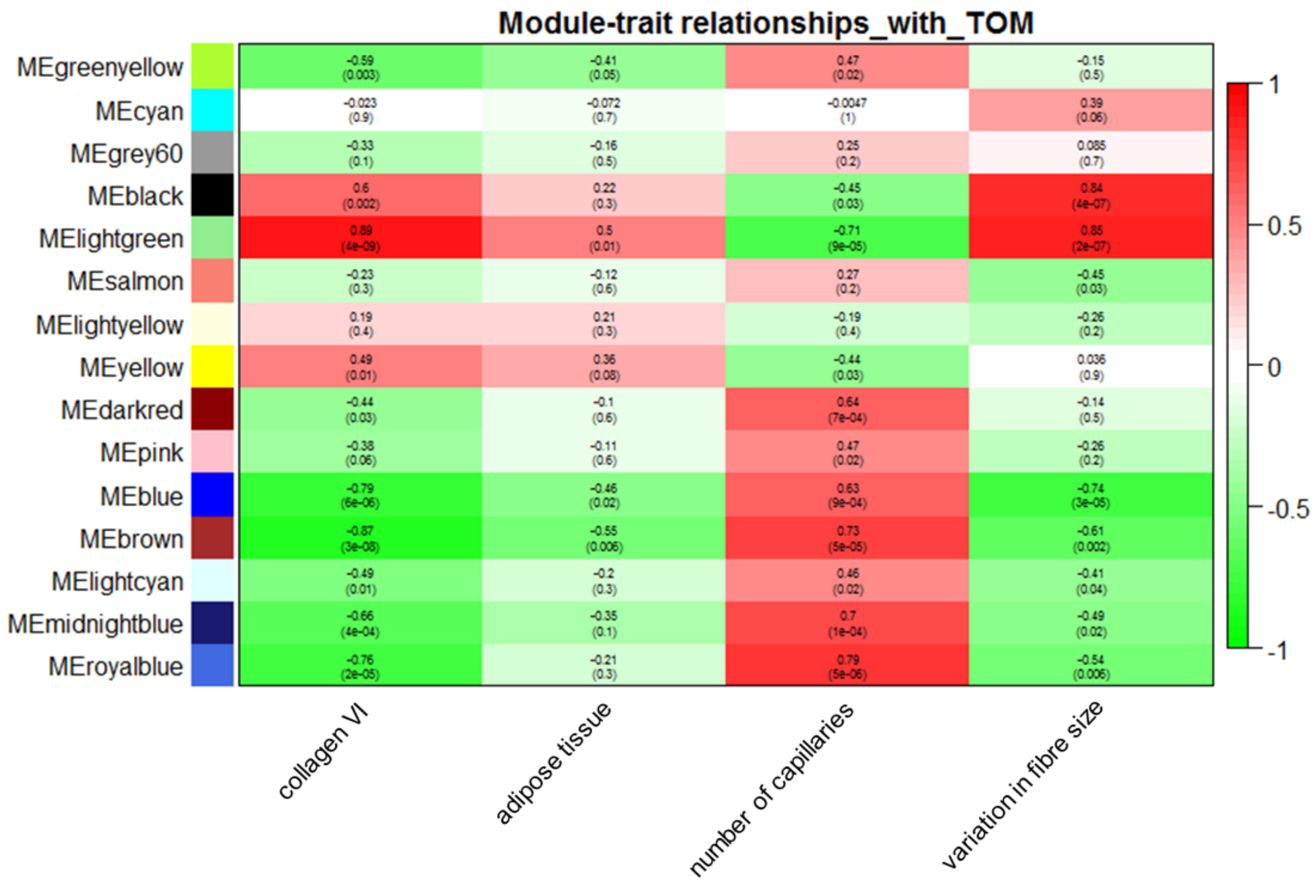

Supplementary Figure S2. Mean gene significance across modules for collagen (**A**), the number of capillaries (**B**) and the coefficient of variation for fibre size (**C**).

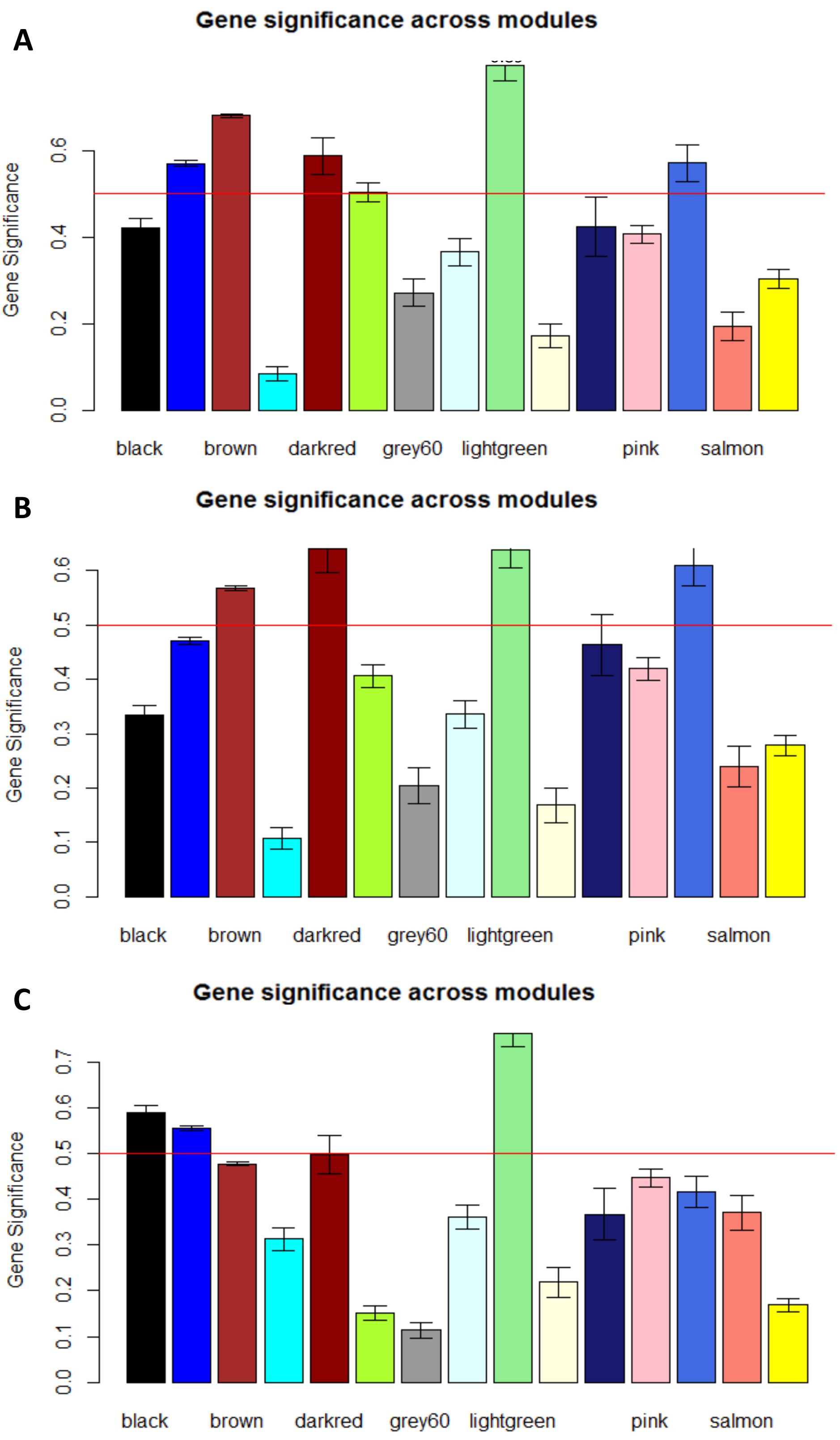

Supplementary Data S2. Probe annotation based on the Galgal5 assembly (see Excel file Supplementary_Data_S2.xlsx).

Supplementary Table S3. Primer sequences and gene ID of the subset of genes used for RT-qPCR validation.

Gene	Gene ID	Forward primer	Reverse primer
CAPN3	423233	CAAACCAGTGCTCATTCCCT	GCCTGACCCACACTGATTTT
CAV3	378796	GAAAGGCAGCTACACCACCT	GTAGATGCGGCTGACACACT
COL6A3	396548	AGCCCAAAGTGACCTACACG	GCAGATGTCCATGACATTTTCAG
FN1	396133	CACAAACACCAACGTCAACT	GTTTGGATGGTAATCGTGGCAG
LRSAM1	417265	AGGTCCTCAATGTGAAAGGCA	TTGCAGAGGAACTGCTGAATG
MYH15	395534	TTCAAGCAAACCCAGCCCTA	GATTTTTCAGCAACCGGGTG
MYH1B	374069	AGGAGCTGTCCAATGTCAACCTC	GCAGAAGAAAGCAACAGAGGGTTC
MYH1E	427788	CCAAATTCCGCAAGATCCAAC	CTTATGCCACTTTGTTGTCACGAC
MYH1F	768566	AGCTGTCCAATGTCAACCTTTCC	TGCCTCAGGTCACACTTTAGC
MYOCD	427790	GTCTGAGCACTCCTTGCTGATT	CGTTGTTCATGGAATGCGG
PDGFRα	395509	GAGTCACAAAAGCCGTGGACAT	TGTTTTCTCACAGGACCGCTC
PPP1R3A	101747406	GGCAAACAACGACGACAAAAAC	TTCTTCCTTGCTGCTTGGTGC
